# Patients’ and Clinicians’ Perceived Trust in Internet-of-Things Systems to Support Asthma Self-management: Qualitative Interview Study

**DOI:** 10.2196/24127

**Published:** 2021-07-16

**Authors:** Chi Yan Hui, Brian McKinstry, Olivia Fulton, Mark Buchner, Hilary Pinnock

**Affiliations:** 1 Asthma UK Centre for Applied Research, Usher Institute Edinburgh United Kingdom; 2 Usher Institute, The University of Edinburgh Edinburgh United Kingdom; 3 Patient Advisory Group, Asthma UK Centre for Applied Research, Usher Institute, University of Edinburgh Edinburgh United Kingdom; 4 Tactuum Ltd Glasgow United Kingdom

**Keywords:** asthma, self-management, telehealth, internet-of-thing, trust

## Abstract

**Background:**

Asthma affects 235 million people worldwide. Supported self-management, including an action plan agreed with clinicians, improves asthma outcomes. Internet-of-things (IoT) systems with artificial intelligence (AI) can provide customized support for a range of self-management functions, but trust is vital to encourage patients’ adoption of such systems. Many models for understanding trust exist, some explicitly designed for eHealth, but no studies have used these models to explore trust in the context of using IoT systems to support asthma self-management.

**Objective:**

In this study, we aim to use the McKnight model to explore the functionality, helpfulness, and reliability domains of patients’ and clinicians’ trust in IoT systems to deliver the 14 components of self-management support defined by the PRISMS (Practical Reviews in Self-Management Support) taxonomy.

**Methods:**

We used *think-aloud* techniques in semistructured interviews to explore the views of patients and clinicians. Patients were recruited from research registers and social media and purposively sampled to include a range of ages, genders, action plan ownership, asthma duration, hospital admissions, and experience with mobile apps. Clinicians (primary, secondary, and community-based) were recruited from professional networks. Interviews were transcribed verbatim, and thematic analysis was used to explore perceptions of the functionality, helpfulness, and reliability of IoT features to support components of supported self-management.

**Results:**

A total of 12 patients and 12 clinicians were interviewed. Regarding perceived functionality, most patients considered that an IoT system had functionality that could support a broad range of self-management tasks. They wanted a system to provide customized advice involving AI. With regard to perceived helpfulness, they considered that IoT systems could usefully provide integrated support for a number of recognized components of self-management support. In terms of perceived reliability, they believed they could rely on the system to log their asthma condition and provide preset action plan advice triggered by their logs. However, they were less confident that the system could operate continuously and without errors in providing advice. They were not confident that AI could generate new advice or reach diagnostic conclusions without the interpretation of their trusted clinicians. Clinicians wanted clinical evidence before trusting the system.

**Conclusions:**

IoT systems including AI were regarded as offering potentially helpful functionality in mediating the action plans developed with a trusted clinician, although our technologically adept participants were not yet ready to trust AI to generate novel advice. Research is needed to ensure that technological capability does not outstrip the trust of individuals using it.

## Introduction

### Background

Asthma is a variable long-term condition that affects 235 million people worldwide [1]. In everyday life, patients decide how to maintain control of their asthma and what to do if their condition worsens. When they are unsure what to do, they contact their health care advisor; within the UK health care system, this would normally be the general practitioner (GP) or primary care asthma nurse. Supported self-management of asthma has many components [2] but specifically includes provision by the patients’ usual health care professional of a personalized action plan summarizing agreed decisions (eg, medication adjustment or emergency strategies) [3-5].

With the increasing availability of sensors and improved coverage of wireless networks, an internet-of-things (IoT) system has the capability to observe patients’ status and medication use and support self-management. Devices have the intelligence to perform a task independently or connect to other sensory networks, platforms, and mobile phones to perform multiple tasks. Artificial intelligence (AI) may be *narrow* (artificial narrow intelligence: systems that interact with users based on a set of planned rules) or can *mimic*, *equal*, or ultimately *surpass* human intelligence to create new ways to interact with users (described as artificial general intelligence or artificial superintelligence, respectively) [6].

The IoT has been used to support clinical management in a range of contexts (eg, asthma, diabetes, and hypertension), with examples including diagnosis, remote monitoring, remote consultation, self-management, emergency care, and home rehabilitation [7-13]. Traditional trust between patients and their clinicians is associated with improved medication adherence and health outcomes [14-18] and can be harnessed to encourage adoption and continued use of digital health systems [19-21]. Underpinning this is a gradual shift in trust from the clinician to technology.

### The Concept of Trust

The concept of trust is *elusive* [22] but is typically illustrated as a relationship between 2 agents (a trustor and a trustee) [23]. Terms such as *confidence*, *have faith in*, and *believe in* are commonly used, and in the health care context, multiple attributes have been summarized broadly as “The belief that a doctor is working in the patient’s best interest” [14]. The term *e-trust* has been used to describe the trust between a human agent (eg, patient, clinician, or health carer) and a digital artifact agent (eg, whether it can achieve a given goal) [24]. However, in an IoT system there may also be *trust* among artifact agents; for example, an AI system may rely on (or *trust*) the technical specifications of a smart device and system to collect and transfer accurate data on which to base advice to the user.

In the context of supported self-management, patients are the core users of digital health services such as health information websites, web-based consultations, or online support groups. Patients adopt telehealth for many reasons, such as personal, technological, institutional, and legislative, but in the decision of delegating a specific task to an intelligent system, a fundamental factor is whether the patient trusts that the system can fulfill their expectations. Models of e-trust have defined multiple factors required for the trustor to decide to trust a digital health system broadly classified as follows [25-30]:

- Personal factors such as altruism, ease of use, self-efficacy, sociodemographic characteristics, usefulness, recommendation by others, fair use of data, and cost.
- Technological factors such as customization, interoperability, and data privacy.
- Institutional factors such as ability (or not) to improve communication with their clinician, professional training, the accuracy of the information provided to the clinical service, service provider’s reputation, the organization’s nature, or business model.
- These might be added to *legislative factors* in the context of health care, as (for example) medical device registration requires evidence of technological performance, effectiveness, and safety, which demonstrates that a product is worthy of trust [31-34].

The McKnight model [35], in comparison with e-trust models, is based on an interpersonal trust model between the human agent and the digital artifact agents and sees trust in technology as task specific (aligned with the Castelfranchi and Falcone [23] cognitive trust model for human agents [36]). This model conceptualizes three dimensions of task-specific trust in technology: functionality, helpfulness, and reliability. In the context of supporting asthma self-management, *functionality* is how patients and clinicians believe an IoT system has the features and capability to accomplish a range of self-management tasks. *Helpfulness* is the degree to which patients and clinicians believe an IoT system can provide an adequate, responsive, and useful aid to support their asthma self-management tasks and decisions. *Reliability* is whether patients and clinicians believe an IoT system can operate continuously and properly to support tasks.

### Trust in the Context of Digital Support for Self-management

Although there are many trust models [20,37], including some in eHealth [29,30,38], no studies have used existing models explicitly to explore trust in using IoT systems to support asthma self-management. The McKnight trust model is task specific, enabling a comprehensive investigation of the app features and various device combinations of the IoT system as opposed to examining the digital health system as a *black box*. Therefore, using asthma as an example, we aim to use the McKnight trust model to explore the domains of trust beliefs between patients or clinicians and IoT systems in the context of the PRISMS (Practical Reviews in Self-Management Support) taxonomy, a framework defining components of self-management support in long-term conditions [2].

## Methods

### Ethical Considerations

The study was conducted between May 2019 and January 2020 with the approval of the London Fulham Research Ethics Committee (ref: 19/LO/0703), sponsored by the University of Edinburgh and the National Health Service (NHS) Lothian (Academic and Clinical Central Office for Research and Development) and funded by the Chief Scientist Office/Asthma UK Innovation Grant (ref: CSO-AUK-2018-03). All participants provided informed consent before the interviews.

### Design

We used semistructured interviews to explore patients’ and clinicians’ trust in using IoT systems to support asthma self-management. Purposive sampling (see *Purposive Sampling* section) continued until we achieved data saturation with regard to our aim; we estimated from previous studies that this would be 12 patients and 12 clinicians [39].

### Patient Recruitment

We recruited people (aged ≥16 years) with *active asthma* (defined as a physician diagnosis of asthma and at least one asthma treatment prescribed in the previous year [40]) in the United Kingdom. We wanted to explore the perceived trust between patients and technology, so we excluded children and adolescents as the involvement of a parent or guardian would have added an additional person to the interactions. We recruited patients through volunteer databases (Scottish Health Research Register [41], Register for Asthma Research [42], Asthma UK volunteer database, and social media of Asthma UK and Asthma UK for Applied Research).

Potential participants were invited to register their interest on our recruitment webpage, which provided an information sheet. They were asked to confirm their eligibility (diagnosed with asthma by their GP, ≥16 years, and living in the United Kingdom), provide their demographics, and give us consent to contact them to complete registration.

### Purposive Sampling

From the information provided, we purposively sampled patients to achieve maximum diversity of perceptions about the use of technology to support self-management. Sampling was based on the following criteria:

- Age range (16-25 years, 26-45 years, 46-65 years, and ≥65 years)
- Ownership of action plan (or not)
- Duration of asthma (diagnosed within <6 months, 6-12 months, 1-10 years, or >10 years)
- Admission to hospital in the past 12 months (or not)
- App download experience (ie, can download apps by themselves, have asked someone to download apps for them, or have never downloaded an app)

### Clinicians’ Recruitment

We recruited primary, secondary, and tertiary care clinicians who provided routine care for children or adults with asthma. We posted advertisements in the newsletter and social media of the NHS Research Scotland Primary Care Network and professional bodies such as the Primary Care Respiratory Society [43]. We also invited individual clinicians known to have an interest in asthma and technology.

### Data Collection

We conducted in-depth semistructured interviews with patients to understand their perceived use of self-management support features and specifically explore their perceived trust in using IoT systems to support their self-management. The potential features we explored were from our previous work and the scope of commercially available devices. We provided images of smart devices and data (Multimedia Appendix 1) and asked patients to design a personalized IoT system incorporating the features they thought would help them live with asthma. We used *think-aloud* techniques to explore their trust (or not) in using the IoT system they had created to support their self-management. Clinicians were asked to formulate IoT systems that would support self-management and the care they provide for people with asthma and explored their trust in the features and the IoT system (see Multimedia Appendix 2 for the topic guide).

### Data Synthesis and Analysis

Interviews were digitally recorded, transcribed, and analyzed using NVivo version 12 (QSR International) [44]. We used the McKnight trust model [35] to categorize patients’ and clinicians’ perceptions of their trust in the functionality, helpfulness, and reliability of using IoT systems to support asthma self-management.

We used a framework analysis [45], creating a matrix of self-management support features against perceptions of the McKnight trust model (or not) expressed by patients and clinicians. All interview data related to trust were extracted into the matrix and aligned with the features to which they referred. To increase applicability to other long-term conditions and because the perceived trust domains (functionality, helpfulness, and reliability) in the McKnight model are task specific, we mapped the perceptions of trust to components used to support self-management in long-term conditions, as described in the Practical Systematic Review of Self-Management Support taxonomy for long-term conditions [2]. We were alert to other trust-related themes that did not fit the matrix either because they did not reflect the domains of *functionality, helpfulness, and reliability* or because they were overarching rather than task-related.

A research team member (CYH) coded 1 patient and 1 clinician interview, which was then reviewed by another researcher (HP). The 2 researchers discussed their decisions and standardized the coding for the rest of the transcriptions. CYH coded all the data related to perceived trust (or not). HP reviewed the matrix for quality control.

### Reflexivity and Interpretation

CYH has research expertise in exploring user preferences for asthma apps and academic interest in developing IoT systems to support asthma self-management. She discussed the coding and interpretation of results with the study team members from different backgrounds and experiences, including GPs, a patient, and a technology developer, to ensure a broad interpretation.

## Results

### Participants

#### Patients

From 362 expressions of interest (268, 74% women), we purposively sampled 12 (3.3%) patients with a range of ages, gender, and action plan ownership for interviews (Textbox 1). The resultant maximum variation sample included more women (8 women and 4 men). None had been diagnosed with asthma for less than a year, and all were confident in their ability to download an app without asking for help.

**Patients’ and professionals’ demographics.**

**Patients (N=12)**

- Age (years): spread across 4 age groups from teenage or young adults to ≥65 years: 16-25 years (n=3), 26-45 years (n=2), 46-65 years (n=3), and ≥65 years (n=4).
- Gender: 8 women and 4 men.
- Ownership of an asthma action plan: only 4 had been given a written action plan. Of the 8 who did not have an action plan, 5 had been “told what to do.” Of the 5 participants who had been “told what to do,” 2 were aged 46-65 years, and 3 were ≥65 years.
- Duration of asthma: 8 (4 men) had had asthma for more than 10 years; none were newly diagnosed.
- Hospital admissions in the previous 12 months: only 4 had had a hospital admission in the previous year, 3 of whom were still under a specialist clinic. None of the male participants had had an admission.
- Experience in using apps: all the participants were confident to download an app by themselves.

**Clinicians (N=12)**

- Primary care clinicians, n=4 (2 general practitioners [GPs] and 2 asthma nurses).
  Gender: 1 man and 3 women.
  Practice experience: GPs with 8 years’ experience; asthma nurses with 20 years’ experience. GPs had research experience in digital health for patients with asthma.
  Technology experience: asthma nurses had experience in using remote telemonitoring for hypertension.
- Secondary care clinicians, n=4 (1 respiratory consultant and 3 respiratory pediatricians).
  Gender: 1 man and 3 women.
  Practice experience: respiratory consultant: diagnostics, asthma management, and severe asthma care; respiratory pediatricians: asthma management in a range of asthma severities.
  Technology experience: 1 had used an asthma app, 1 uses smart inhalers in their service and research, and 2 had research experience in asthma technology.
- Pharmacists, n=4 (1 hospital pharmacist and 3 primary care support pharmacists or prescribing advisors).
  Gender: 1 man and 3 women.
  Practice experience: 1-14 years’ experience in reviewing asthma medications.
  Technology experience: all used web-based repeat prescriptions services; 1 developed an asthma app.

#### Clinicians

We recruited 12 UK clinicians (GPs, asthma nurses, pharmacists, consultant chest physicians, and respiratory pediatricians) who provided care for people with asthma. Most had experience using technologies such as smart inhalers, mobile apps, and SMS text messages to support respiratory patients in their practices or hospitals.

### Overview of Results

Perceptions related to the 3 domains of the McKnight model of task-specific trust in technology (functionality, helpfulness, and reliability [35]) are synthesized below. Multimedia Appendix 3 lists the perceived trust in functionality, helpfulness, and reliability in IoT features related to generic long-term conditions or asthma self-management tasks. Finally, we considered the overarching domain of trust in data security, which was clearly important to our participants, reflecting not only the properties of the technology but also the context within which it was implemented.

### Perceived Functionality of IoT Components to Support Self-management

Most patients perceived that the IoT had functionality that could well support a range of self-management tasks (Multimedia Appendix 3 lists examples of tasks that participants trusted the IoT to deliver). This belief was often based on past technological experiences:

I do use technology to control my asthma, so I keep copies of my peak flows and I can do charts on my laptop so, when I am deteriorating and I end up in hospital, I can take this with me and show them that obviously it’s happened over a period of days. And I use alarms on my phone as well so I can wake up and have my medication because I have to have four-hourly nebulisers as well at the minute to control it.

P6, 16-25 years, female

I tend to put a reminder on my phone so I can have the one (asthma review) in a year’s time, but it is a bit of effort.

P10, 16-25 years, female

Some patients perceived that the IoT system could have the functionality to support how they lived with asthma, although these features were not yet available in the market:

I think if there was something similar [to energy saving tips in a smart home] on the app where you’re using the app but it gives you a tip each day that you know, “air pollution could be a trigger for your asthma” or “washing can be a trigger for asthma”, then that might give you some additional information.

P7, 46-65 years, male

I think kind of mindfulness breathing exercises you can find on, like, YouTube. If it was, like, breathing exercises to assess the asthma, it might be the sort of thing I might try once and see what I thought of it and if I thought it was useful I might try it again.

P10, 16-25 years, female

Some clinicians perceived that the IoT had the functionality to engage patients to look after their asthma and support self-management. They believed (in the future) systems could transfer patients’ manual or auto logs to a health care professional for review or flag up when inhaler medication needed to be replenished. In contrast, others doubted whether technology could change patients’ behavior:

There isn’t an app that I’m aware of that can link with the GP systems. So if that is possible from a technology perspective, inputting how much they’re using and there’s a log then of when they have their new prescription, and then that app then talks to the GP system it can flag when they get to a certain level and order a repeat, I think that’s perfectly feasible.

HCP2, pharmacist in hospital, female

I think patients either are physically active or they’re not, and the app’s not going to make them physically active if they’re not.

HCP5, consultant chest physician, female

In the last few months in my pharmacy we’ve introduced...well, we always had online ordering but there wasn’t huge engagement with it but we’ve introduced an app-based system for ordering. A younger population who is ordering things like asthma inhalers and contraceptive pills and so on have really engaged with that actually quite well.

HCP7, pharmacist in practice, male

### Perceived Helpfulness of Supporting Components of Self-management

Most patients had a perception that IoT systems could provide a useful service to provide integrated support for a number of recognized components of self-management support [39] (Multimedia Appendix 3 lists some examples of tasks that participants thought would be helpful for the IoT to provide).

They wanted IoT systems to log data about their asthma symptoms, peak flow, medication use, inhaler technique, indoor or outdoor environmental data, activity intensity, and weight, and perceived it would be helpful if these data could be collected effortlessly, such as a voice assistant asking about their asthma (eg, “Good morning! Did your asthma disturb your sleep last night?”) or automatic data collection from wearable devices or environmental sensors in their living areas. Some specific ways in which they thought an IoT system would be useful if they could help them look after their asthma by providing customized alerts and advice were the following:

- Identifying unusual asthma symptoms or peak flows and automatically providing customized information about their asthma and advice on medication adjustment and follow-up actions (suggesting and counting the number of rescue puffs to be taken in an emergency, calling medical help)
- Alerting them if their inhaler technique was incorrect
- Detecting unusual use of rescue inhalers to help them identify what triggered their asthma
- Reminding them to comply with their preventer inhaler

In addition, they thought that an IoT system would be helpful in supporting their communication with clinicians. Most participants believed it would be helpful to be able to ask quick questions or arrange follow-up consultations with clinicians via text, WhatsApp, or email and then be able to share their data with clinicians to assess their asthma status. Some patients thought objective evidence from logs would help explain their asthma to their friends or senior colleagues at work:

I’ve missed so many events in my life because of my asthma and I think it’s difficult to say to someone. I think if you had this medical evidence behind you, they’d understand without you having to explain it.

P6, 16-25 years, female

I think particularly my parents. I live in a flat on my own and if for whatever reason during the night I was suddenly puffing my blue inhaler multiple times, I’d almost want a warning siren to be sent to my parents just in case I’m really struggling.

P12, 26-45 years, female

A patient who had had a recent hospital admission thought it would be helpful to automatically share their asthma logs with the emergency department and share test reports between different hospitals to prevent treatment delay. Some patients described how they panicked when they were very short of breath and could lose track of how many puffs of their reliever inhaler they had taken in a short period. A system that counted the doses of reliever inhaler they had taken and warned them in real time about overdose would be a helpful safety net. Patients with multimorbidity wanted the system to integrate information from different health care specialists about all their treatments and provide medication advice to reduce the side effects of different drugs.

Most clinicians agreed that receiving data about peak flow and symptoms would help them assess asthma status in reviews, but also highlighted the benefits of an IoT system that could transfer objective data on incorrect or correct inhaler technique and medication use to help assess the adherence and suitability of the inhaler device:

Because very often patients don’t remember to bring their inhaler with them so it’s difficult to always test when they’re in the clinic. So if you’re being alerted to that, when they’re doing it at home, then that’s perfect, because if you ask a patient are you doing it right, they always say “yes”.

HCP1, GP, female

If I’ve got some hard data on their peak flow and their symptoms over the last couple of months, and their adherence, that gives me a much better idea of what I need to do with them, so that’s incredibly helpful for me.

HCP5, consultant chest physician, female

### Perceived Reliability

Patients and clinicians discussed reliability—whether they trusted the IoT system would operate continuously and without error—in two contexts: logging data and providing advice.

#### Logging Data

Some patients observed that a system that logged data (such as coughing, sleep disturbance, and medication use) automatically *in the background* would reduce missing data. They believed that smart peak flow meters and smart inhalers could reliably capture data, although there were caveats. Some patients did not always carry these devices with them or had more than one reliever inhaler in use (at home, at work, and in the car), and a reliable system would need to accommodate these behaviors. Some patients suggested that a voice assistant was easier to use, but others raised concerns about its accuracy. Most clinicians agreed that automatic logs are more accurate, as they reduce human error:

If it can capture most things, like obviously in the air it’s cold or there’s pollen or there’s pollution, I could probably trust it quite a bit, that, because it’s solid data that’s already captured in other places.

P1, 46-65 years, female

I think it (an IoT system) might be more accurate as well than say if I did it (logging) myself.

P11, 16-25 years, female

I know some people say that sometimes they [patients] come in and they sit in the waiting room and they’re filling in the results.

HCP4, prescribing support pharmacist in practice, female

I suppose adding technology in to it might make it more accurate and take out the human error and that.

HCP10, GP, male

#### Providing Advice

Most patients believed that the system could accurately highlight the advice on an agreed action plan when their condition was getting worse but were skeptical that the system could safely generate new advice. They would trust the system to reliably prompt an alarm when their condition worsened or if they took their inhaler incorrectly, identify environmental triggers and recommend avoidance, and remind them of the actions suggested on an agreed action plan. In contrast, all patients preferred their clinician to interpret the data and decide on new advice. Similarly, patients did not believe that the system could take *human factors* (such as the impact of psychological or emotional context) into account when reaching a diagnosis. Clinicians were also comfortable with an IoT-based early warning system to alert patients to seek further assistance when their condition worsened but considered the automatic generation of new advice as an *unproven route*. They also cited the importance of personal relationships. They accepted that AI may be used to generate new intelligent advice to patients in the future but would need evidence to prove clinical accuracy before trusting in its reliability:

Well, again it goes through two stages, so if I’m really bad, maybe a message to say I’m really bad and...probably notice my wife first (to make decisions) and then the health care professional (to arrange follow up actions)...I wouldn’t want that to trigger an appointment with a health care professional.

P4, 26-45 years, male

Like if it was suggesting changes to me, that feels more like the time that I would actually have to have a conversation with the GP or nurse rather than my phone triggering stuff like that.

P12, 26-45 years, female

So if there’s some sort of really intelligent system that can work out how to do an asthma action plan for somebody based on intelligent peak flow monitoring and intelligent looking at the symptoms and all of that, great, but until we’ve got that we need a human being I think to sit with the patient and make an asthma action plan. Because even as an experienced clinician it can be quite challenging sometimes because you have to know quite a lot about asthma to do them.

HCP5, consultant chest physician, female

I don’t think we’re at a stage where a system can advise patients. I would be a little bit nervous about it. I’d have to have proof that that actually works because I think I would recognize that in my practice I establish a relationship with a patient. A machine advice, automated advice doesn’t necessarily understand that.

HCP11, pediatrician, male

### Trust in Data Security

Privacy of personal data was a strong overarching theme that emerged in the interviews. Although clearly relevant to trust but not task specific (as in the McKnight trust model), patients were found to accept the health services to implement IoT systems if they knew how their data would be used. Attitudes varied, with a patient suggesting they were not concerned about data security, whereas another explained that they were not happy to use a voice assistant because it was connected to the cloud service. Most patients and clinicians wanted to use SMS text messages or emails for follow-up questions, although both suggested that the General Data Protection Regulation was a barrier to adopting these services in the NHS. Clinicians balanced the data privacy risk and the helpfulness of the services and thought that explaining to patients about the use of their data and having their consent was a pragmatic approach, as opposed to blocking the service completely:

That (Email communication) would be useful sometimes, but they (health care professionals) wouldn’t do it, so I don’t really know...(the clinicians) they’d be worried about that (spam in email), same with text messages and WhatsApp...It might work from my side, but I don’t think it would work from their side.

P5, >65 years, male

The NHS contract can be difficult, with regards to GDPR and so on, so nearly everything is done via phone, and if you can’t speak to an actual person, we don’t routinely leave messages and so on.

HCP7, pharmacist in practice, male

This is personal data but it’s only about your health condition. So in one way I wouldn’t be that worried about that so much because actually that’s just about one condition that actually you want to make sure that people know about so that you actually get treated properly.

HCP4, prescribing support pharmacist in practice, female

## Discussion

### Principal Findings

Most patients believed IoT systems to be functional and helpful in supporting a broad range of self-management tasks, but they raised some concerns about reliability. They believed IoT systems could collect their data accurately from devices, check for incorrect inhaler techniques, and advise them on treatment options based on the thresholds and actions agreed with clinicians (eg, in an action plan) and customized to their situation. However, they doubted whether the system could interpret their data to generate novel advice or reach diagnostic conclusions. They would want to check with a health care professional for reassurance and *human* advice before acting on AI-determined actions. Most patients’ beliefs resonated with those of the clinicians. Before trusting and adopting AI-developed advice, clinicians wanted evidence to reassure them about accuracy. Pragmatic approaches are required to deliver services based on the requirements of the General Data Protection Regulation. Our study did not find a diversity of views among different ages and genders, possibly because all participants had experience with technology and were the end users of similar NHS asthma care services in the United Kingdom. Racial biases, sociocultural norms, and an understanding of AI are other potential factors that need to be considered when developing IoT-supported services applicable to diverse communities.

### Strength and Limitations

We explored perceived trust in IoT systems from the perspective of patients and clinicians; however, there are some limitations. First, patients and clinicians based their opinions on their past experience of existing technologies and arrangements within current health care services. Our clinician participants were interested in technology and asthma, which will have influenced their opinions that were based on their experience, personal interest, age, and gender. The views from these groups of participants may not apply to users with limited access to technology or lack of experience with digital options. Real-life experiences with an IoT system may have generated new themes. However, our findings represent perceived expectations from patients and clinicians and can therefore inform future IoT system design and underpin further investigation. Second, because of time and resource limitations, we did not interview children (patients aged ≤16 years) and their parents, although we included experienced pediatricians to explore some of the issues from their perspective. Third, we could not recruit patients who were newly diagnosed (0-1 year) with asthma who may have had specific requirements, although our experienced asthma patients provided some feedback on their needs or expectations when they were newly diagnosed. Fourth, all of our participants were confident in using technology such as social media, web information, voice assistants, and activity trackers. Hence, participants may be biased in assessing perceived functionality, helpfulness, and reliability because of their past use of technologies. However, their real-life experience enabled them to give examples of IoT features that they considered trustworthy (or not) from personal experience. Finally, the McKnight domains used in this study were limited to perceived functionality, helpfulness, and reliability; other domains such as perceived ease of use, perceived value, and the source of the recommendation (eg, an app recommended by a clinician that the patient believes understands their asthma may engender more trust in technology than an app recommended by a clinician that the patient does not know or trust) [21,46] may also be important to the perceived trust in the asthma self-management IoT system.

### Interpretation in Relation to the Published Literature

Our findings show that patients and clinicians both recognized the potential of IoT systems to provide a range of customized support for self-management, which they believed would help them look after their asthma. They trusted smart devices to observe their status accurately and had confidence that the system could trigger advice previously agreed with the clinician when they experienced unusual asthma symptoms or reduced peak flows. They found it acceptable for systems to detect errors, correct inhaler techniques, and address noncompliance to medication. These functions imply that IoT systems can be trusted to include AI that can learn about an individual’s asthma throughout time and provide advice based on a set of rules. However, neither patients nor clinicians trusted the IoT system to mimic clinicians’ intelligence and create new self-management advice, preferring a human check to reassure that the AI advice was applicable to the individual before deciding what to do. This resonates with the findings of a recent review on AI clinical interventions in the context of other long-term conditions, such as depression, weight, nutrition, limb pain, and smoking cessation management [47]. People trusted a customized system including elements that imitated human-human (patient-clinician) interactions and provided easy communication channels between the patient and clinician. Furthermore, the involvement of clinicians was pivotal to encourage patients’ adoption and adherence to digitized self-management [21].

Technically, patients and clinicians are reluctant to move from using *narrow* intelligence that follows preset rules (artificial narrow intelligence) to general (artificial general intelligence) or superintelligence (artificial superintelligence) in which the system initiates rules. Although there are high-level guiding principles [48,49] and governance recommendations [50] to ensure that future AI designs are ethically and technically trustworthy [51] (eg, to ensure the use of AI is fair, is transparent, and meets universal human values), they focus on the trust between AI and the community. Few have explicitly considered trust between AI and individual patients in the context of supported self-management.

Patients are not yet ready to transfer their trust from the clinician (a human) they know to an IoT system (a machine) generating self-management advice through AI. In the traditional self-management model, the GP or asthma nurse assesses the patient’s condition and agrees with self-management advice in a face-to-face consultation. In the new IoT self-management model, the app interface, smart devices, or lifelike robots (in the near future) have the responsibility to sense the patients’ condition, which replaces the clinicians’ intelligence in giving self-management advice to patients. The decision process is an impenetrable *black box* for patients and clinicians. In contrast, clinicians in the traditional model can discuss options with the patient and consider aspects such as patients’ mood, personality, self-management habits, and experiences so that the final decision is (relatively) transparent. This may be a reason many patients trust that AI-based IoT systems can record their asthma condition better than themselves, but none have shifted their trust from the clinician to the AI in terms of issuing new advice.

From e-commerce literature, we know that it is possible to shift people’s trust from a known person, organization, or shop to an electronic service related to the known entity [30,52,53] or recommended by the known person, organization, or shop [54]. Iterative interaction with an automated system or lifelike robot can build up trust for first-time users who are curious about new systems and robots but struggle to use them in their daily lives [55,56]. In the health care context, studies of apps and e-consultations have suggested the potential to transfer trust from a physical health care service (eg, appointment booking and monitoring physiological parameters or activity after discharge from hospital) to an app [57-59]. However, to encourage clinicians to recommend an AI system to patients, strong clinical evidence is required to earn their trust. Currently, there is little evidence in the context of asthma self-management to reassure clinicians or patients.

### Conclusions

Introducing IoT systems involving advice from AI to support self-management requires more than just functionality that can deliver tasks users regard as helpful. There is a need to increase the trust of users in the reliability of systems as AI moves from the currently acceptable *narrow* intelligence directed by clinician-determined action plans to a future in which advice is generated by the IoT system. Our technologically adept participants were not yet ready for this step; research is needed to ensure that technological capability does not outstrip the trust of the individuals using it.

## Appendix

Multimedia Appendix 1 Images of smart devices and data.

Multimedia Appendix 2 Topic guide.

Multimedia Appendix 3 Perceived trust table.

## Abbreviations

AI: artificial intelligence
GP: general practitioner
IoT: internet-of-things
NHS: National Health Service
PRISMS: Practical Reviews in Self-Management Support
