# Supplementary material for: Patients’ and Clinicians’ Perceived Trust in Internet-of-Things Systems to Support Asthma Self-management: Qualitative Interview Study
Source: JMIR Mhealth Uhealth. 2021 Jul 16;9(7):e24127. doi: 10.2196/24127 (PMC8325078; doi:10.2196/24127)
Supplement: Multimedia Appendix 1 [file mhealth_v9i7e24127_app1.pdf]

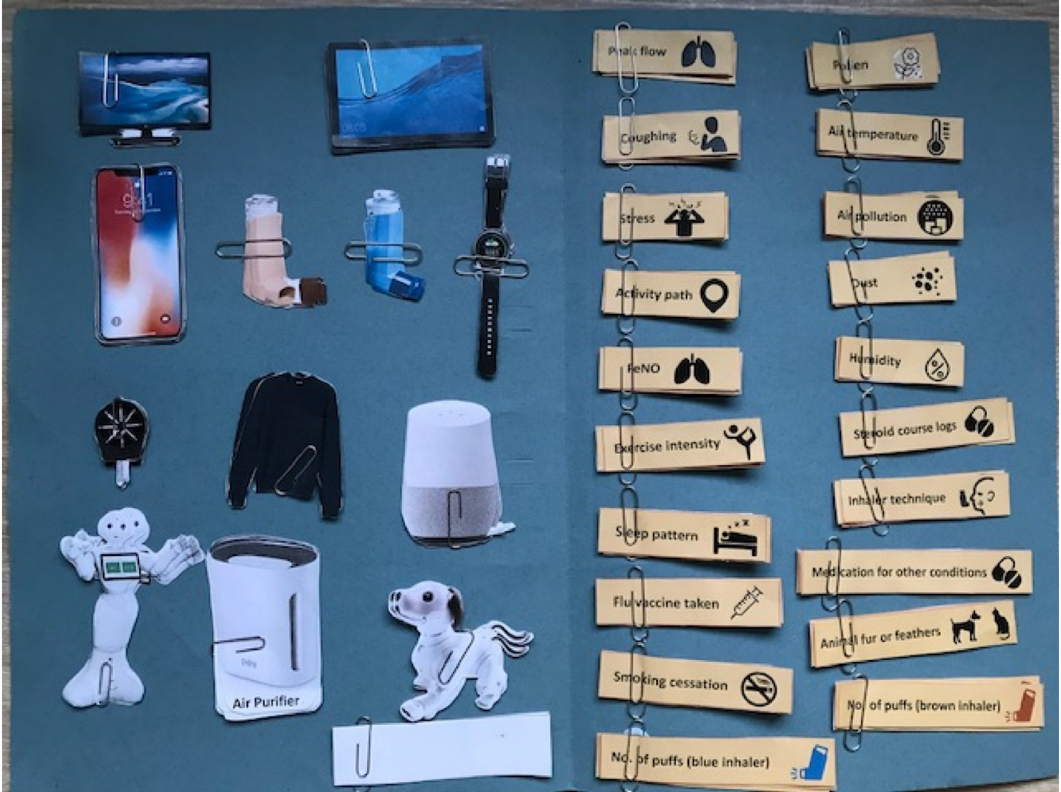

Peak flow
Coughing
Stress
Activity path
FeNO
Exercise intensity
Sleep pattern
Flu vaccine taken
Smoking cessation
No. of puffs (blue inhaler)
Pollen
Air temperature
Air pollution
Dust
Humidity
Steroid course logs
Inhaler technique
Medication for other conditions
Animal fur or feathers
No. of puffs (brown inhaler)
Air Purifier
