# Supplementary material for: Patients’ and Clinicians’ Perceived Trust in Internet-of-Things Systems to Support Asthma Self-management: Qualitative Interview Study
Source: JMIR Mhealth Uhealth. 2021 Jul 16;9(7):e24127. doi: 10.2196/24127 (PMC8325078; doi:10.2196/24127)
Supplement: Multimedia Appendix 3 [file mhealth_v9i7e24127_app3.pdf]

Patients' and Clinicians' Perceived Trust in Internet-of-Things Systems to Support Asthma Self-management: Qualitative Interview Study

CY Hui, B McKinstry, O Fulton, M Buchner, H Pinnock (correspondence: hilary.pinnock@ed.ac.uk)

Multimedia Appendix 3: Trust in IoT systems to support self-management related to perceptions of functionality, helpfulness, and reliability

**Components of self-management support** **(from PRISMS taxonomy [2])**	**Functionality** **Self-management tasks that participants believed the IoT system was capable of performing**	**Helpfulness** **IoT based tasks that participants believed would be useful and contribute to the self-management their asthma**	**Reliability** **Tasks that participants believed the IoT system could perform continuously and properly continue to support self-management. ***	**The degree of AI needed to fulfil patients' expectation, and overall perceived trust in IoT systems**
Information about asthma and available resources	• Customising asthma information to individual patients. E.g. linking to online video for correct inhaler technique	• Interactive verbal communication (e.g. AI assistant) with the IoT system to seek customised and reputable information such as medication information, general information about asthma, medical information, new approaches to managing asthma.	Some patients were not sure if the IoT system would work accurately to suggest personalised asthma information based on observing their asthma conditions and needs over time.	A system that provide customised asthma information to patients via communication between the intelligent agent and patients<br>[Artificial narrow intelligence]
Regular clinical review, communication with healthcare professionals, text communication	Providing:<br>• Regular consultation reminders<br>• Online and video-consultations<br>• Whatsapp, text and email with clinicians	• Text messages to remind about consultations<br>• (For patients with hearing problems) Text message to replace phone communication with clinicians.<br>• Online/teleconsultations for quick questions about asthma to save the time it takes for a visit<br>• On-line booking for appointments	Most patients believed the system could accurately prompt an alarm to remind about a consultation<br>Most patients would trust the system to support online consultations and text communications.	An online consultation system that provides online video meeting and text service to reach clinician with an alarm to remind their appointment.<br>[No artificial intelligence needed]
Monitoring condition with feedback	• Sending log reminders<br>• Automatically logging asthma symptoms, peak flow, medication<br>• Logging exercise intensity with smart devices	• Visualising logs on graphs to work out when their asthma is getting worse; what is affecting and triggering their asthma<br>• Automatically detect and flag reliever use to prevent overuse and prompt remedial action.	Most patients believed the system could be relied on to set reminders and to collect monitoring data.<br>They believed the system could display data on a graph and warn them when their condition was getting worsened correctly.	An intelligent early warning system to warn patients before their asthma is getting worsen.<br>[Artificial narrow intelligence]

		• Sharing logs with clinicians to avoid unnecessary reviews if all is well<br>• Sharing logs and test results with different clinicians to prevent treatment delay<br>• Home monitoring of condition to reduce stays in hospital		
Provision of action plan, training for everyday activities	• Storing scans of the paper-based action plans on phone.<br>• Monitoring environmental data<br>• Linking with indoor and outdoor sensors to provide air pollution and high pollen dashboards and alerts	• A digitised action plan providing advice according to the agreed actions for the green, amber and red zone to give prompt advice on:<br>○ Medication adjustment<br>○ Emergency relief actions<br>○ Calling for urgent medical attention<br>• Integrate advice from different clinicians and for other conditions to reduce medication side effects<br>• Giving alert when local air pollution/ pollen count is high, to help to plan the day	Most patients believed the system could remind them of the actions suggested on an agreed action plan and alert them when their monitoring data fell below the threshold set on the action plan by the clinician.<br>However, they doubted if the system could take decisions how to diagnose or treat their condition without a clinicians' reviews.<br>Some believed the system could reliably support them and their clinicians to identify triggers by highlighting data on a graph.<br>Most of the patients believed the could rely on the system to provide air quality alerts.	An intelligent patient decision supporting system to show various personal asthma data and environmental data on an app/web dashboard. Also, to provide everyday advice to patient base on the existing asthma action plan template and rules (threshold and actions). Clinicians will need to review the data and give new rules when needed. [Artificial narrow intelligence]
Practical support with adherence	• Providing medication adherence reminders on smart inhaler<br>• Monitoring use of inhalers and ordering repeat prescriptions when needed<br>• Flu vaccine reminder	• Checking medication compliance and alerting when forgotten<br>• Indicating the number of doses left in the inhaler device and prompting ordering a repeat prescription when running low<br>• Alerting when flu vaccine is available; and online links for booking an injection appointment	Most patients believed the system could reliably monitor adherence to medication, and count the dose left in an inhaler, but most wanted to be alerted before the system re-ordered the medication. They were not sure how, but they believed it would be more accurate than their own judgments of when a canister was empty (e.g. shaking it)<br>They believed the system could reliably monitor stocks of vaccine	An intelligent medication reminder and counting system that links to existing repeat prescription system. When the medication is low, the system will alert patients a few weeks before the medication is running out. Patients to decide to process a re-ordering or not. [Artificial narrow intelligence]<br>A flu vaccine reminder system that link to the existing practice online system.<br>[No artificial intelligence needed]
Provision of equipment	• Linking to smart inhaler, and peak flow meters<br>• Using voice assistant, smart watches to form a sensory network	• Smart devices and sensors that capture data silently to save time and avoid missing data.	Most patients believed smart devices and sensors could collect data correctly. Some, with experience of voice assistants, did not believe it could work properly (both mis-understanding their speech and mistaking what they wanted). Some believed smart fabric could detect their data correctly but	A real-time sensory network with a mechanism to check with patients to iteratively learn what the data mean to individual patients.<br>[Artificial narrow intelligence]

			unsure how it worked. One patient believed an injection chip (under skin) could reliably detect data.	
Provision of easy access to support when needed	• Provision of a panic button<br>• Enabling remote consultations, including transfer of data	• Sending automated text messages to friend or emergency service if struggling to breathe.	Most patients believed the IoT system could transfer their text message to the person they wanted to know about the emergency.	An emergency text support system to send message to a particular person or emergency when patient press the button.<br>[No artificial intelligence needed]
Training for self-management activities	• Automatic monitoring of inhaler technique (smart inhalers)<br>• Linking to videos to correct inhaler technique.	• Alerting if inhaler technique is poor and assist self-learning of correct inhaler technique.	Most patients believed the system could detect and alert their incorrect inhaler technique by using the camera on the phone or intelligent detection methods on a smart inhaler, though they were unsure how it worked.	An intelligent inhaler technique checking device or system that can detect incorrect inhaler technique and teach patients the correctly inhaler technique with video or refer them to clinicians.<br>[Artificial narrow intelligence]
Training for psychological strategies, social support,	• Breathing exercises video/on app/online class.<br>• Notifying friends/family about their asthma,	• Teaching breathing exercises to help calm and relieve asthma symptoms.<br>• Sending an auto reply message to a friend when they have been admitted to the hospital.	Some patients believed the system could reliably teach breathing exercises.<br>They also believed the system can send messages just like usual SMS and Whatsapp services.	An activity tracking system to provide information such as breathing exercise and logs; with an auto reply service set on their existing text service when the patients have admitted to the hospital.<br>[No artificial intelligence needed]
Lifestyle advice and support	• Customising fitness training for asthma.	• Cross referencing exercise intensity, asthma symptoms and use of rescue medication to suggest fitness training.<br>• Weight watching programmes to help losing weight if needed	Most patients with fitness tracking devices and apps, fitness and weight watching support believe the system could reliably monitor their exercise intensity, weight, asthma symptoms and use of rescue medication and show the data on a graph correctly to support their decisions.	A system that provide customised fitness training to patients via communication between the intelligent agent and patients<br>[Artificial narrow intelligence]

Note:
*Most of the patients generally believed automatic data logging by sensors and devices can work well and provided accurate data to support different tasks. However, it needed to have proper consents with patients to make sure they agreed with the use of data.
